# Ampulla of Vater Metastasis From Colon Cancer Presenting As Obstructive Jaundice

**DOI:** 10.7759/cureus.115155

**Published:** 2026-08-25

**Authors:** Palash Gupta, Kuppusamy Senthamizhselvan, Pazhanivel Mohan, G Ramkumar, Nachiappa Ganesh Rajesh

**Affiliations:** 1 Department of Medical Gastroenterology, Jawaharlal Institute of Postgraduate Medical Education and Research, Puducherry, IND; 2 Department of Radiodiagnosis, Jawaharlal Institute of Postgraduate Medical Education and Research, Puducherry, IND; 3 Department of Pathology, Jawaharlal Institute of Postgraduate Medical Education and Research, Puducherry, IND

**Keywords:** ampulla of vater, biopsy, colonic neoplasms, drainage, immunohistochemistry

## Abstract

A woman in her late 60s presented with obstructive jaundice. She had mucinous adenocarcinoma of the colon six months ago and underwent surgery followed by adjuvant chemotherapy. A computed tomography scan revealed a prominent ampulla with duodenal wall thickening, dilated intrahepatic biliary radicles, bile ducts, and the main pancreatic duct. Duodenoscopy revealed a bulky, friable, and ulcerated ampulla of Vater (AOV) with partial luminal narrowing. The biopsy and immunohistochemistry (IHC) confirmed adenocarcinoma of colonic origin metastasizing to the AOV. She underwent an endoscopic retrograde cholangiogram and biliary drainage, and best supportive care before dying of her illness. Metastatic involvement of AOV is extremely rare. This case reiterates the need to consider AOV metastasis in the differential diagnosis of obstructive jaundice, especially in patients with a history of malignancy. It also underscores the role of IHC to differentiate AOV primary malignancy from metastasis.

## Introduction

Colorectal cancer (CRC) is the third most common cancer globally [1] and the fourth most common cancer in India [2]. Despite advances in surgical treatment and chemotherapy, approximately 4%-8% of patients develop local recurrence, and distant metastases may occur in the liver, lungs, peritoneum, or ovaries [3]. The ampulla of Vater (AOV) is a small anatomical structure in the second part of the duodenum where the common bile duct and pancreatic duct open into the intestine, and secondary tumors involving the AOV are rare. The primary tumors reported to metastasize to the AOV most commonly arise from the breast and kidneys [4]. Metastatic CRC involving the duodenal bulb and second part of the duodenum has been reported previously [5,6]. In contrast, metastasis to the AOV from CRC has been reported only three times to date [7-9]. We report a rare case of mucinous adenocarcinoma of the colon in an older woman with metastasis to the AOV, presenting with obstructive jaundice due to impaired bile flow into the duodenum.

## Case presentation

A woman in her late 60s presented with jaundice, abdominal pain, and pruritus for the past four weeks. She had nausea, decreased appetite, and weight loss of 6 kg. She did not have fever, abdominal distension, or bleeding tendencies. She was diagnosed with colon cancer (mucinous adenocarcinoma, pT3N2bM0) six months ago. She underwent laparoscopic right hemicolectomy and ileotransverse anastomosis followed by six cycles of adjuvant chemotherapy comprising capecitabine and oxaliplatin. She had no comorbidities. Her past medical history and family history were unremarkable, with no known cases of gastrointestinal malignancies. On general physical examination, she was ill-nourished, icteric, and had left supraclavicular lymphadenopathy. Abdominal examination revealed an enlarged gall bladder. Examinations of the respiratory, cardiovascular, and nervous systems revealed no abnormalities. Blood investigations revealed anemia, cholestatic jaundice, and elevated carcinoembryonic antigen (Table 1).

**Table 1:** Baseline laboratory investigations

Investigation	Result	Reference range
Hemoglobin	87 g/L	120-150 g/L
White blood cell count	8.4 × 10⁹/L	4.5-11 × 10⁹/L
Platelet count	235 × 10⁹/L	150-450 × 10⁹/L
Total serum bilirubin	7.14 mg/dL	0.2-1.2 mg/dL
Direct serum bilirubin	3.90 mg/dL	0.0-0.3 mg/dL
Alkaline phosphatase	532 IU/L	30-120 IU/L
Gamma-glutamyl transferase	588 IU/L	0-38 IU/L
Aspartate aminotransferase	91 IU/L	0-35 IU/L
Alanine aminotransferase	67 IU/L	0-35 IU/L
Carcinoembryonic antigen	4.6 ng/mL	0-3 ng/mL

**Figure 1:**
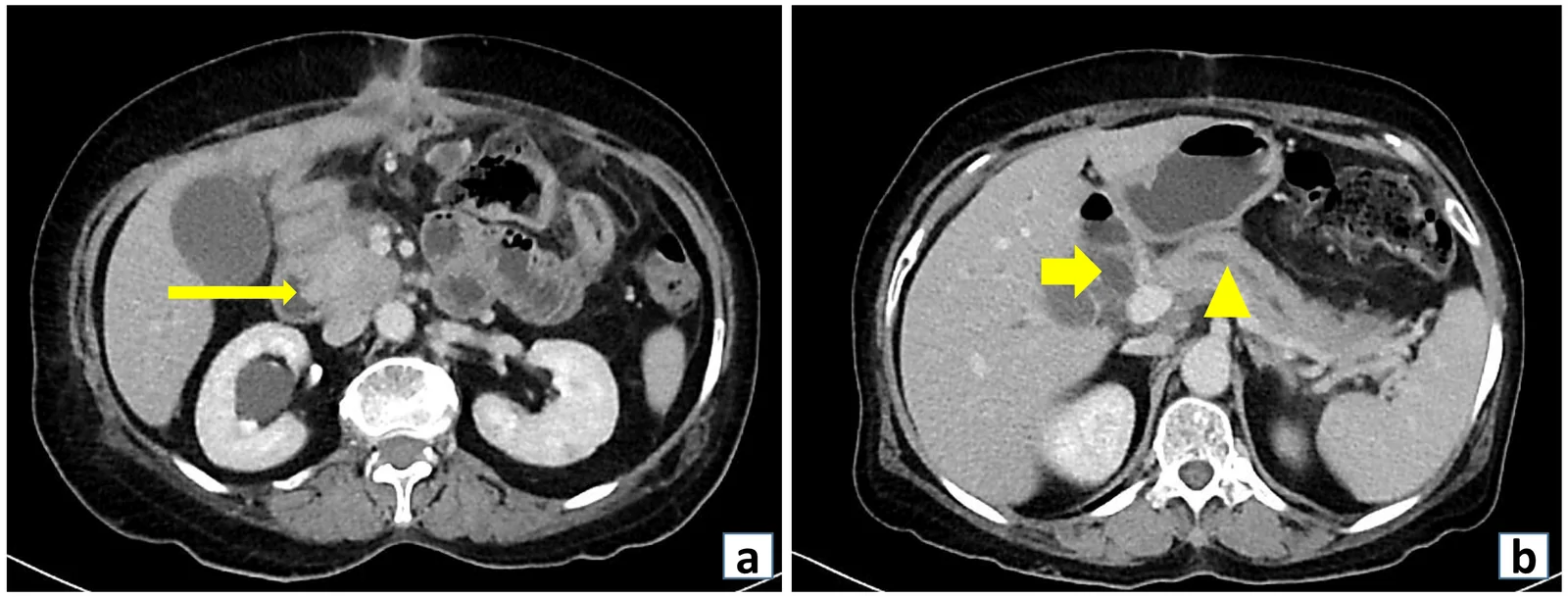
Computed tomography scan of the abdomen with (a) slender arrow pointing to a prominent ampulla and (b) arrowhead pointing to a dilated main pancreatic duct and bold arrow pointing to a dilated common bile duct

Computed tomography (CT) scan of the abdomen showed a prominent AOV with duodenal thickening, dilated intrahepatic biliary radicles, common bile duct, and main pancreatic duct (Figures 1a, 1b).

**Figure 2:**
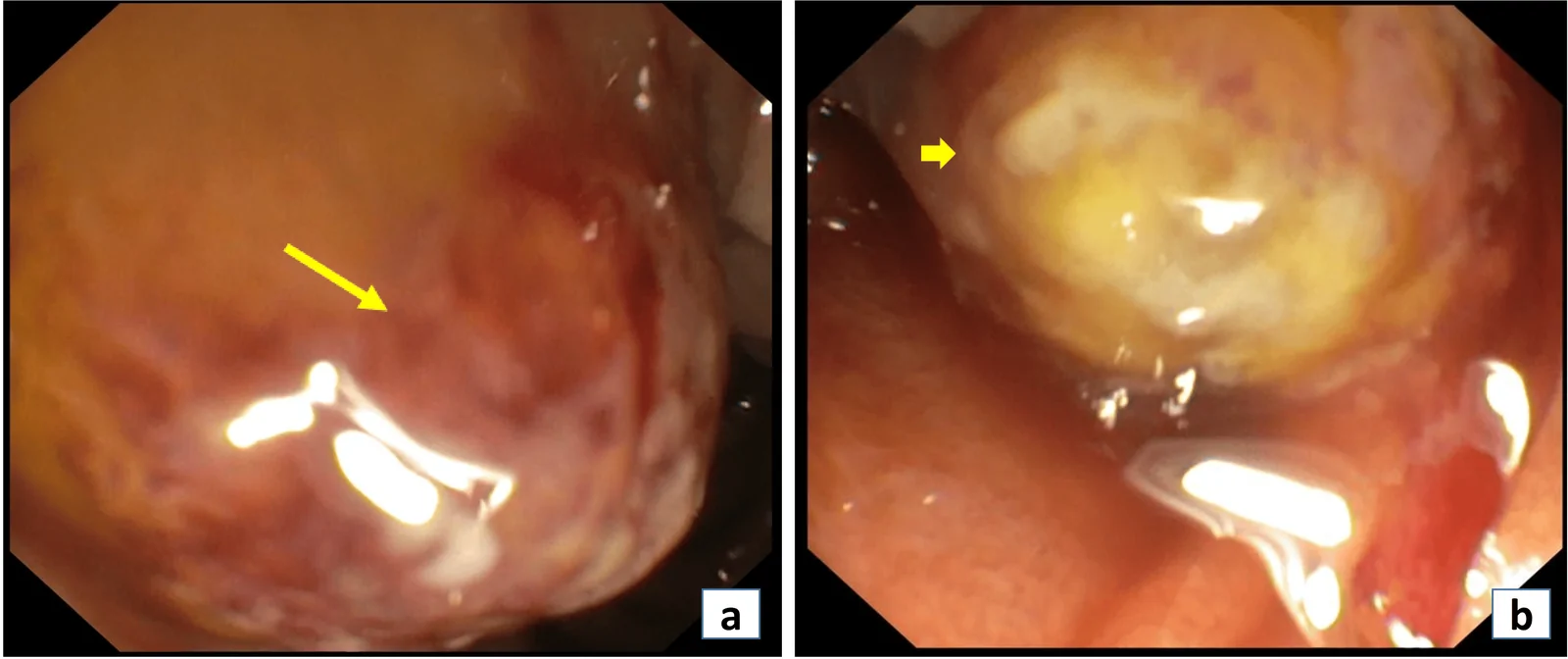
Duodenoscopy image with (a) slender arrow showing a bulky ampulla of Vater and (b) bold arrow showing ulceration around the papillary orifice

Duodenoscopy revealed a bulky, ulcerated AoV (Figures 2a, 2b).

**Figure 3:**
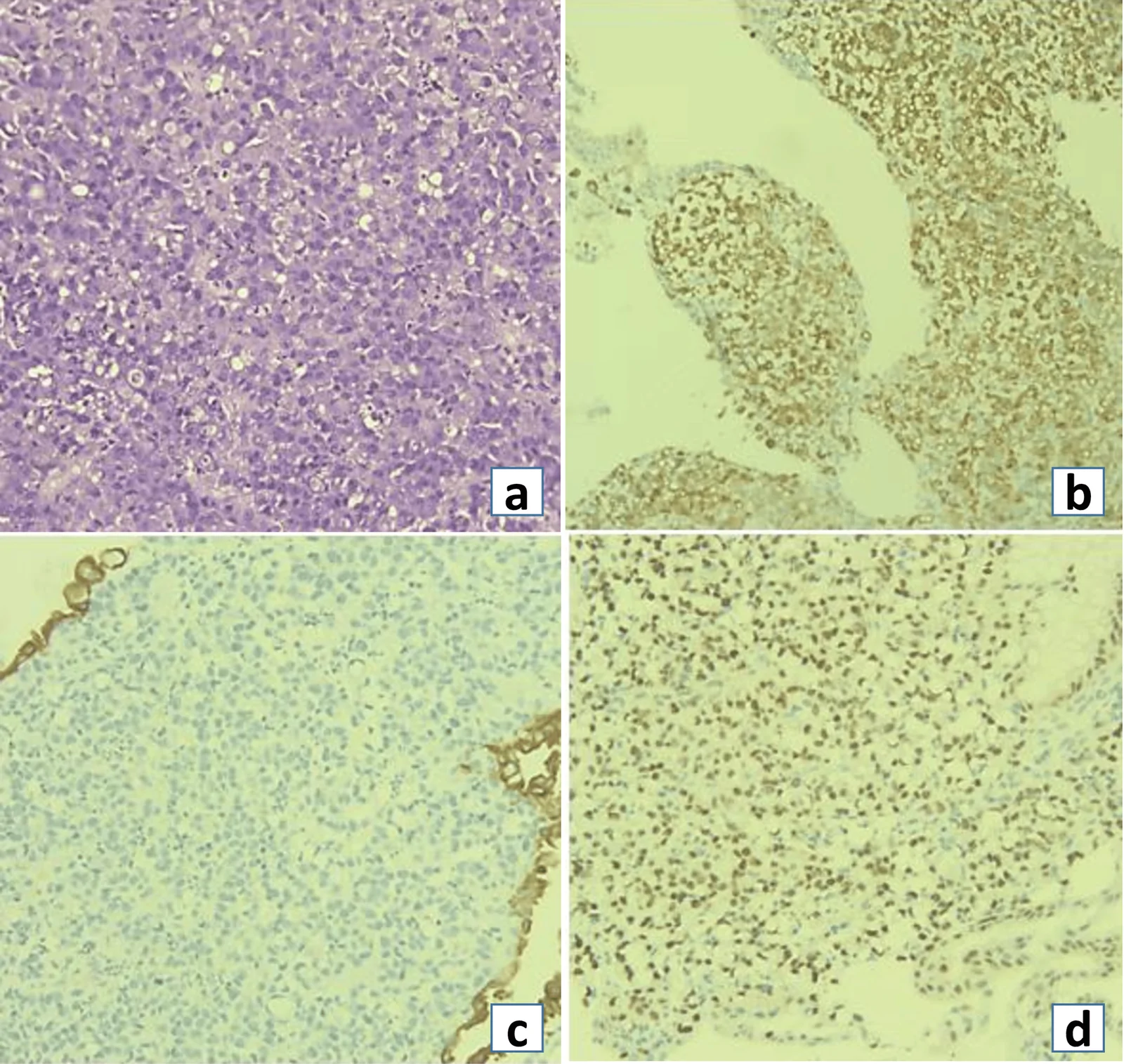
Photomicrographs of (a) biopsy from ampulla showing sheets of tumor cells, with abundant mucin-filled vacuoles (hematoxylin and eosin stain, 20× magnification). Immunohistochemistry with (b) CK7 highlighting the lining epithelium and being negative in tumor cells, (c) CK20 highlighting tumor cells and being negative in the lining epithelium, and (d) CDX2 highlighting both lining epithelium and tumor cells CK7: cytokeratin 7; CK20: cytokeratin 20; CDX2: caudal-type homeobox 2

Biopsy revealed a well-differentiated adenocarcinoma with mucin production. On immunohistochemistry (IHC), cytokeratin 20 (CK20) and caudal-type homeobox 2 (CDX2) were strongly positive, whereas cytokeratin 7 (CK7) was negative, suggesting a colonic origin of the metastasis (Figures 3a-3d).

Fine needle aspiration cytology from the left supraclavicular lymph node also showed metastatic adenocarcinoma. We considered the following differentials while evaluating this case: primary malignancy of the AOV, pancreatic head cancer, distal cholangiocarcinoma, duodenal malignancy, and nodal metastasis causing extrinsic compression of bile ducts. The CT findings of simultaneous dilatation of the common bile duct and pancreatic duct ("double duct sign") with abrupt narrowing at the ampullary region strongly suggested an ampullary-level obstruction. Furthermore, the duodenoscopy findings prompted us to consider a primary malignancy of the AOV. However, the histopathology and IHC markers, namely CK7 negativity, CK20, and CDX2 positivity, confirmed AOV metastasis of colonic origin.

She underwent an endoscopic retrograde cholangiogram (ERC), and a 10-Fr, 7-cm straight, plastic stent was placed for biliary drainage. She had a poor performance status (Eastern Cooperative Oncology Group score of 3) and hence received the best supportive care [10]. Following ERC and stenting, the patient reported gradual improvement in jaundice and cholestasis. However, the patient's overall performance status deteriorated gradually, and despite our care, the patient expired eight weeks later.

## Discussion

Metastasis from CRC occurs via the portal venous system, lymphatics, or peritoneal fluid. Mucinous tumors have a more aggressive peritoneal spread due to mucin production, which facilitates this process [11]. The liver is typically the first and most common site of metastasis in colon cancer, but when dissemination is multifocal, the thorax is most often additionally involved [3]. The third and fourth parts of the duodenum are involved in direct spread from the colon cancer. Sarocchi et al., in their review, found that the primary tumors causing ampullary metastasis are breast, kidney, malignant melanoma, ovary, endometrium, cervix, and esophagus [4]. Brahmbhatt et al. reported a case of recurrent adenocarcinoma of the colon metastasizing to the duodenum, presenting as partial gastric outlet obstruction after two years [5]. Chaudhary et al. reported a case of ileocecal adenocarcinoma who underwent right hemicolectomy three years ago and then presented with a central mesenteric mass infiltrating into the second part of the duodenum [6]. Iwamuro et al. described a case of adenocarcinoma of the sigmoid colon, with metastatic lesions located in the duodenal bulb. Each lesion appeared as a submucosal tumor with an ulcer at the center [12]. Diamond et al. described three cases of primary right colonic tumors metastasizing to the duodenum [13]. The metastatic involvement of the AOV is exceedingly rare. Alfonso et al., in their case series on duodenal metastasis, reported a case of AOV metastasis from a primary arising in the cecum [7]. Taş et al. reported synchronous malignancies from the caecum and AOV in a patient who underwent endoscopic ampullectomy [8]. Samara et al. reported a case of AOV metastasis incidentally diagnosed during surveillance two years after the initial diagnosis of rectal cancer. The patient underwent pancreaticoduodenectomy and received adjuvant chemotherapy [9].

The rarity of AOV metastasis is due to the absence of direct portal venous and lymphatic drainage from the colon, the small size of the ampulla, and preferential sequestration of tumor cells in the liver and lungs. The clinical presentation of AOV metastasis is usually nonspecific, with symptoms limited to the primary tumor. Rarely, it can present with gastrointestinal bleeding, obstructive jaundice, and abdominal pain. The median time to AOV metastasis after primary tumor diagnosis is 4.8 years; however, synchronous metastasis can also occur [4]. In our case, it was diagnosed six months later. Endoscopy might reveal ulceration, polypoidal mass, or nodularity. These features are indistinguishable from primary ampullary malignancy. Histopathology and IHC markers help to differentiate primary malignancy from metastasis. Primary ampullary adenocarcinomas can be subdivided into intestinal-type and pancreatobiliary-type based on differentiation. The IHC patterns are also distinct: CK20+/CDX2+/CK7-/MUC2+ favors intestinal differentiation, whereas CK7+/MUC1+/CDX2-/CK7- favors pancreatobiliary differentiation. The metastasis usually bears markers of the primary tumor. It is also noted that pancreatobiliary is a more aggressive type associated with more perineural and nodal invasion and decreased overall survival. The presence of Special AT-rich sequence-binding protein 2, along with strongly positive CDX2, favors a colorectal origin [14,15].

Ampullary tumors can be benign adenomas or adenocarcinoma. In suspected cases, non-invasive imaging such as CT or MRI can be performed to localize the lesion. CT will be more useful for distant metastasis, whereas soft-tissue involvement and the extent of bile duct or pancreatic duct involvement are better seen on MRI. Endoscopic ultrasound can be used for T-staging and fine needle aspiration of the targeted lesion. Duodenoscopy gives direct visualization of the lesion and sample collection for pathological diagnosis. ERC can be used for defining intraductal tumor infiltration and biliary drainage before surgery or chemotherapy [16].

The management approach includes surgical resection of primary and metastatic lesions with curative intent in selected patients, and debulking palliative surgery for symptom relief in others. The surgical approach carries significant morbidity and mortality risk. Biliary drainage via ERC and stenting is a minimally invasive strategy that provides symptomatic relief for patients. Systemic therapies, including cytotoxic chemotherapy, targeted molecular agents, and immunotherapy, form the cornerstone of treatment for most patients. Oxaliplatin- or irinotecan-based fluoropyrimidine regimens (FOLFOX, CAPOX, and FOLFIRI) and intensified multi-agent regimens (FOLFOXIRI) are first-line therapies [17]. The addition of vascular endothelial growth factor pathway inhibitors (bevacizumab, aflibercept, or ramucirumab) improves progression-free and overall survival irrespective of the status of the RAS mutations [18]. Cetuximab or panitumumab is recommended for RAS wild-type, left-sided tumors in combination with chemotherapy [19]. Pembrolizumab or nivolumab with or without ipilimumab is standard first-line therapy for microsatellite instability-high and mismatch repair-deficient metastatic CRC [20]. BRAF V600E-mutant disease benefits from encorafenib and cetuximab, whereas the newer options include regorafenib and trifluridine-tipiracil [21].

## Conclusions

In conclusion, AOV metastasis, though extremely rare, must be considered in the differential diagnosis of obstructive jaundice, particularly in patients with malignancy. The clinical, endoscopic, and radiologic findings of AOV metastasis are nonspecific. Histopathology with IHC is essential to distinguish metastatic disease from primary ampullary carcinoma. Management of AOV metastasis is usually palliative. However, early recognition helps us plan appropriate interventions and engage in multidisciplinary decision-making.
